# When Warm Meets Cold: Mixed-Type Autoimmune Hemolytic Anemia Revealing an Underlying Autoimmune Disorder

**DOI:** 10.7759/cureus.101398

**Published:** 2026-01-12

**Authors:** Mukilan S, Dheeraj Jain, Kavitha Ramachandran

**Affiliations:** 1 Department of General Medicine, Indira Gandhi Medical College and Research Institute, Puducherry, IND

**Keywords:** autoimmune hemolytic anemia (aiha), cold agglutinin, coombs test, corticosteroid therapy, direct antiglobulin test, mixed-type autoimmune hemolytic anemia, warm antibody

## Abstract

Autoimmune hemolytic anemia (AIHA) is a rare immune-mediated disorder caused by autoantibodies directed against red blood cell antigens. Mixed-type AIHA, characterized by the coexistence of warm-reactive IgG and cold-reactive complement-fixing antibodies, is uncommon and often diagnostically challenging due to overlapping serologic features and transfusion incompatibility. We report the case of a 40-year-old woman who presented with an acute onset of breathlessness, palpitations, and severe fatigability. She was found to have severe anemia (hemoglobin 2.7 g/dL), reticulocytosis, indirect hyperbilirubinemia, and red cell agglutination on peripheral smear. Both direct and indirect Coombs tests were strongly positive, while antibody screening demonstrated panreactivity with positive autocontrol. The monospecific direct antiglobulin test confirmed positivity for IgG and C3d, establishing the diagnosis of mixed-type AIHA. In view of hemodynamic instability, transfusion with the least-incompatible packed red cells was performed under warming precautions, alongside high-dose intravenous corticosteroids. The patient improved symptomatically, with hemoglobin rising to 8.8 g/dL. Autoimmune workup revealed high-titer antinuclear antibody and anti-SSA positivity, suggesting an evolving systemic autoimmune disorder. Mixed-type AIHA accounts for less than 10% of cases but is associated with significant morbidity due to combined extravascular and complement-mediated hemolysis, often necessitating cautious transfusion of least-incompatible blood in life-threatening anemia and escalation to rituximab or other immunosuppressive agents. This case highlights the diagnostic complexity, therapeutic challenges, and need for long-term autoimmune surveillance in patients with mixed-type AIHA.

## Introduction

Autoimmune hemolytic anemia (AIHA) is an uncommon, immune-mediated disorder characterised by the destruction of red blood cells due to autoantibodies directed against erythrocyte surface antigens, with an estimated incidence of 1.7 to 2.4 cases per 100,000 population per year [1-3]. It is classified into warm, cold, and mixed types according to the temperature at which the antibody reacts with the red cells. Warm AIHA involves IgG antibodies active at 37°C, cold AIHA is mediated by IgM antibodies that fix complement (C3d) at temperatures below 37°C, while mixed-type AIHA exhibits features of both, with simultaneous IgG and C3d positivity. These autoantibodies result in dual-pathway hemolysis, wherein IgG-coated erythrocytes undergo splenic macrophage-mediated extravascular destruction, while cold-reactive IgM antibodies activate complement with C3d deposition, leading to complement-mediated hemolysis predominantly in the liver and intravascular compartment [1,4-7]. Mixed-type AIHA is infrequent and diagnostically challenging due to overlapping serologic features, clinical severity, and transfusion difficulties arising from broad autoantibody reactivity and crossmatching incompatibilities. Failure to recognize mixed-type AIHA may result in inappropriate management, delayed immunosuppression, avoidable transfusion-related complications, and progression of potentially life-threatening anemia. We report a case of mixed-type AIHA in a middle-aged woman who presented with life-threatening anemia necessitating urgent transfusion, ultimately revealing an underlying systemic autoimmune process.

## Case presentation

A 40-year-old female with no known comorbidities presented to the Emergency Medicine Department with an acute onset of breathlessness, associated with palpitations, giddiness, and profound fatigability for one week. There was no history of fever, recent infection, weight loss, night sweats, lymphadenopathy, recent drug intake, prior blood transfusions, or previous episodes of anemia or jaundice. The patient denied cold intolerance, Raynaud phenomenon, acrocyanosis, thromboembolic events, recurrent pregnancy loss, or symptoms suggestive of connective tissue disease, such as photosensitivity, oral ulcers, malar rash, arthritis, alopecia, sicca symptoms, or parotid swelling. On examination, she was tachypneic and tachycardic. A general physical evaluation revealed marked pallor, icterus, bilateral pitting pedal edema, and elevated jugular venous pressure. Cardiovascular auscultation identified a soft, blowing systolic murmur of grade 2/6 intensity over the left upper sternal border. Abdominal examination demonstrated a soft, non-tender splenomegaly, palpable 3 cm below the left costal margin. Respiratory and neurological examinations were within normal limits.

Initial laboratory investigations revealed severe anemia with mild thrombocytopenia. Peripheral smear showed anisocytosis with red cell clumping suggestive of agglutination and occasional spherocytes. A markedly elevated reticulocyte count (13.14%) indicated a compensatory marrow response. Renal function was normal, and liver function tests showed isolated indirect hyperbilirubinemia with preserved transaminase and albumin levels. Table 1 shows her baseline investigations.

**Table 1:** Laboratory investigations PCV: packed cell volume; MCV: mean corpuscular volume; MCH: mean corpuscular hemoglobin; MCHC: mean corpuscular hemoglobin concentration; WBC: white blood cells; AST (SGOT): aspartate aminotransferase (serum glutamic oxaloacetic transaminase); ALT (SGPT): alanine aminotransferase (serum glutamic pyruvic transaminase); ALP: alkaline phosphatase; PT: prothrombin time; INR: International Normalised Ratio; LDH: lactate dehydrogenase; DCT/DAT: direct Coomb's test/direct antiglobulin test; ICT/IAT: indirect Coomb's test/indirect antiglobulin test; ANA(IF): antinuclear antibody (immunoflouresence method); SSA-antibody: Sjögren's-syndrome-related antigen A autoantibodies; urine ACR: urine albumin creatinine ratio; MR: mitral regurgitation; TR: tricuspid regurgitation; LVEF: left ventricular ejection fraction; mg/dL: milligrams per decilitre; mEq/L: milliequivalents per liter; IU/L: International Units per Liter; pg: picogram; fl: femtoliters; cu mm: cubic millimetre; sec: seconds

Paramete	Result	Reference range
Hemoglobin	2.7 g%	11.5-16.5 g% (female)
PCV	8.6%	37-47% (female)
MCV	102 fl	78-98 fl
MCH	32.1 pg	27-32 pg
MCHC	31.4 g%	32-36 g%
WBC	6200/cu mm	4000-11000/cu mm
Platelets	129000/cu mm	150000-450000/cu mm
Reticulocyte count	13.14%	0.5-2%
Sodium	141 mEq/L	135-145 mEq/L
Potassium	4.1 mEq/L	3.5-5.0 mEq/L
Chloride	102 mEq/L	96-105 mEq/L
Urea	20 mg/dl	15-40 mg/dl
Creatinine	0.9 mg/dl	0.7-1.2 mg/dl
Bilirubin (total)	3.5 mg/dl	0.4-1.2 mg/dl
Bilirubin (direct)	0.8 mg/dl	0.1-0.4 mg/dl
Bilirubin (indirect)	2.7 mg/dl	0.3-0.8 mg/dl
Protein (total)	8.3 g/dl	6.3-8.3 g/dl
Albumin	4.1 g/dl	3.5-5.5 g/dl
Globulin	4.2 g/dl	2.5-3.5 g/dl
AST (SGOT)	15 IU/L	5-40 IU/L
ALT (SGPT)	10 IU/L	5-45 IU/L
ALP	40 IU/L	30-125 IU/L
PT (Test)	9.4 sec	11-13.5 sec
PT (Control)	11.8 sec	11-13.5 sec
INR	0.7	0.8-1.2
LDH	347 U/L	125-220 U/L
Vitamin B12	296.58 pg/ml	180-914 pg/ml
Ferritin	269.90 ng/ml	15-200 ng/ml
DCT/DAT (polyspecific)	Positive (++++)	Negative
ICT/IAT	Positive (++++)	Negative
Monospecific DAT	IgG (2+), C3d (3+) - positive	Negative
Autocontrol	Positive (++)	Negative
Antibody screening (3 cell panel)	Pan reactive (++++)	Negative
Antibody identification (11-cell panel)	Pan reactive (++++)	No antibody detected
ANA (IF)	Strongly positive (1:1000), speckled pattern	Negative
SSA-antibody IgG	Positive (>200)	Negative (<20)
Urine ACR	<30 mg/g	<30 mg/g
Ultrasound abdomen and pelvis	Hepatosplenomegaly	Normal
2D echocardiogram	Mild MR, Mild TR, trivial pericardial effusion, LVEF-60%	

Given the clinical picture and laboratory findings, immune-mediated hemolysis was suspected. Both direct and indirect Coombs tests were strongly positive. A provisional diagnosis of mixed AIHA was made. In view of the patient’s symptomatic anemia and hemodynamic instability, transfusion with the least incompatible packed red blood cells was carried out under special precautions in a warm, dark room, and high-dose intravenous methylprednisolone therapy was initiated. Further immunohematologic evaluation with a three-cell antibody screening panel demonstrated panreactivity, indicating the presence of broadly reactive antibodies. To delineate the specificity, an extended antibody identification panel using eleven reagent red cell samples was performed, which also revealed uniform panreactivity. The combination of panreactivity and a positive autocontrol supports the presence of a high-titer autoantibody, rather than a specific alloantibody. The monospecific direct antiglobulin test (DAT) showed strong positivity for both IgG and C3d, confirming the coexistence of warm and cold autoantibodies. These findings are consistent with a diagnosis of mixed-type AIHA. Corticosteroids were continued, and an additional unit of least incompatible packed cells was transfused during her hospital stay.

**Figure 1:**
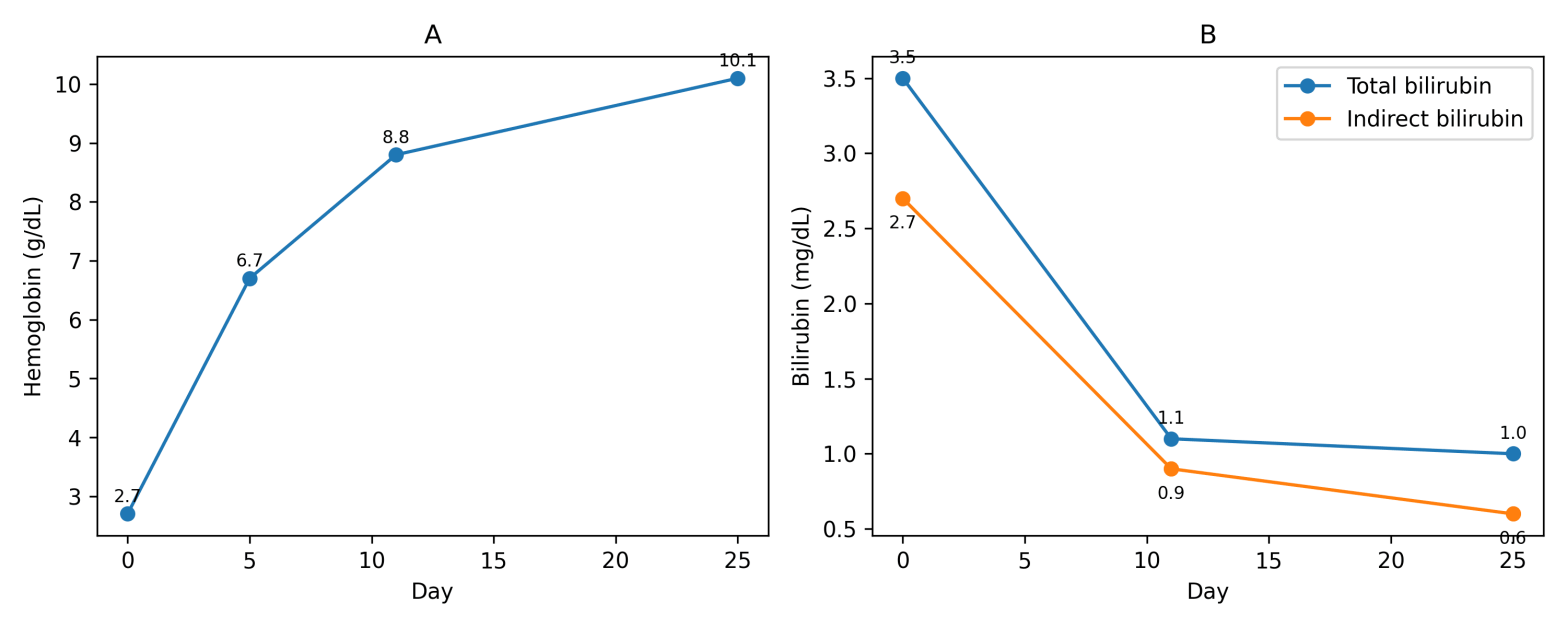
Temporal changes in the patient’s laboratory tests A: Temporal changes in hemoglobin levels. B: Temporal changes in total and indirect bilirubin levels. Serial monitoring demonstrated a sustained rise in hemoglobin and a parallel decline in indirect bilirubin, consistent with effective suppression of immune-mediated hemolysis.

Further evaluation for an underlying autoimmune etiology revealed a strongly positive antinuclear antibody (ANA) titer of 1:1000 with a speckled immunofluorescence pattern. An extended nuclear antibody profile showed positivity for SSA antibodies, while all other autoantibodies were negative. The patient exhibited steady clinical improvement, with resolution of her symptoms and a rise in hemoglobin to 10.1 g/dL. Indirect hyperbilirubinemia also showed a significant reduction, with total bilirubin decreasing to 1 mg/dL. She was discharged in stable condition on a tapering course of oral corticosteroids and advised regular outpatient follow-up for monitoring. A rheumatology consultation was obtained during OPD follow-up. At the time of evaluation, the patient did not fulfill the American College of Rheumatology/European League Against Rheumatism (ACR/EULAR) classification criteria for systemic lupus erythematosus or other defined connective tissue diseases, despite serologic positivity for ANA and anti-SSA antibodies. In the absence of clinical features suggestive of systemic autoimmune disease, disease-specific immunomodulatory therapy was not initiated, and the patient was advised close rheumatologic follow-up for surveillance and early detection of evolving systemic autoimmunity. The temporal changes in laboratory tests of patients during the course of treatment are shown in Figure 1.

## Discussion

Mixed AIHA is a rare entity, the prevalence being around 6.5-8.3% of AIHA cases [8,9]. Compared with previously reported cases, this patient presented with unusually profound anemia, although the pattern of serologic findings and association with systemic autoimmunity were similar to those described in the literature. It is characterised by the coexistence of warm-reactive IgG antibodies and cold-reactive complement-fixing IgM antibodies, reflecting an overlap of immunopathologic mechanisms seen in both warm and cold AIHA subtypes [1,5,7]. Mixed-type AIHA may arise as a primary idiopathic disorder or occur secondarily in association with systemic autoimmune diseases (such as SLE, Sjogren’s syndrome, mixed connective tissue disorder), lymphoproliferative malignancies (such as chronic lymphocytic leukemia and non-Hodgkin lymphoma), infections (e.g., *Mycoplasma pneumoniae*, Epstein-Barr virus), post-transplant immune dysregulation, or drug-induced autoimmunity [10-12]. In some instances, mixed-type presentations may precede the overt manifestation of an underlying systemic disorder by months or even years. In the present case, a strongly positive antinuclear antibody (ANA) with anti-SSA/Ro reactivity suggests a strong evolving autoimmune background, necessitating careful rheumatologic follow-up. The coexistence of warm and cold autoantibodies results in dual-pathway hemolysis, in which two distinct immunologic mechanisms operate. IgG-coated red blood cells are cleared by splenic macrophages via extravascular hemolysis, while cold-reactive IgM antibodies activate complement, leading to C3d deposition and complement-mediated hemolysis in the liver and intravascular compartment [1,5,7]. This leads to rapid and severe anemia, as seen in this case, and often necessitates emergent packed red blood cell transfusion [10,11]. However, transfusion support in mixed AIHA poses significant challenges.

The presence of broad-spectrum autoantibodies interferes with serologic crossmatching, rendering most units incompatible. The decision to transfuse should depend more on the patient’s clinical status and comorbidities (particularly underlying cardiac or pulmonary disease) than on absolute hemoglobin levels. Therefore, while transfusions in AIHA should be deferred whenever feasible, they should not be withheld in cases of severe, symptomatic, and life-threatening anemia, even in the absence of fully compatible units. In such contexts, transfusion with least-incompatible, ABO-negative, Rh-negative, and Kell-matched red blood cell units is warranted following a careful risk-benefit assessment and should be administered under close clinical and transfusion monitoring [13,14]. Measures such as using blood warmers set to 37°C and avoiding cold exposure during transfusion are critical to prevent further hemolysis, particularly in cases with significant cold agglutinin activity. Maintaining a warm ambient environment and minimising patient exposure to low temperatures are equally essential components of supportive care [14,15]. First-line therapy in mixed-type AIHA consists of high-dose corticosteroids, typically prednisone at 1-1.5 mg/kg/day or intravenous methylprednisolone, which modulate the immune response by reducing antibody production and macrophage-mediated red cell clearance [13,14,16]. While warm AIHA generally shows a 75-80% initial response to steroids, the cold component is notably less responsive, often requiring unacceptably high doses to achieve clinical control [13-15]. In rapidly progressive or life-threatening hemolysis, pulse-dose IV methylprednisolone (250-1000 mg/day) may be administered for 3 days [13,16]. However, cure rates remain low (~20-30%), and early escalation to second-line agents is often necessary in partial or non-responders [13,16]. Among these, rituximab, a monoclonal antibody targeting CD20, has become the preferred second-line therapy across AIHA subtypes, including mixed-type, with overall response rates reaching 80-90% and a median time to response of three to six weeks [10,13,16,17]. 

Combination regimens with bendamustine or fludarabine have shown added efficacy but carry increased hematologic toxicity [18,19]. In cases where rituximab is contraindicated or ineffective, conventional immunosuppressants like azathioprine and cyclophosphamide may be used, although responses are variable and limited by adverse effects including myelosuppression, urotoxicity, and secondary malignancy [10,13,16]. Splenectomy, once standard for steroid-refractory warm AIHA, plays a diminished role in mixed-type disease due to the hepatic predominance of complement-mediated hemolysis [10]. Emerging therapies for refractory warm AIHA include neonatal Fc receptor (FcRn) blockers (e.g., efgartigimod) and spleen tyrosine kinase (Syk) inhibitors (e.g., fostamatinib), which target IgG-mediated mechanisms. Agents such as Bruton tyrosine kinase (BTK) inhibitors (e.g., ibrutinib), proteasome inhibitors (e.g., bortezomib), and complement inhibitors (e.g., sutimlimab, eculizumab), though established in cold agglutinin disease, are being cautiously explored in mixed-type AIHA, particularly when cold agglutinin activity or complement-mediated hemolysis predominates; however, clinical evidence in mixed-type AIHA is currently sparse and largely extrapolated from cold or warm subtype studies [13,16]. In secondary AIHA, management should be directed not only at controlling hemolysis but also at treating the underlying disease. In autoimmune disorders such as systemic lupus erythematosus (SLE), corticosteroids remain first-line therapy and often achieve remission of hemolysis while simultaneously controlling systemic disease activity. Hydroxychloroquine is recommended as background therapy in SLE and may contribute to long-term disease control and steroid sparing. In patients with steroid-refractory or steroid-dependent disease, rituximab is effective in both SLE-associated AIHA and idiopathic AIHA by depleting autoreactive B cells.

Other immunosuppressive agents such as azathioprine, mycophenolate mofetil, cyclophosphamide, and methotrexate may be selected based on the severity of systemic involvement, organ manifestations, and patient tolerance [20]. In AIHA secondary to lymphoproliferative disorders, treatment is primarily directed toward the underlying malignancy, often resulting in improvement of hemolysis [12]. Infection-associated AIHA requires appropriate antimicrobial therapy in addition to supportive care, while drug-induced AIHA necessitates prompt discontinuation of the offending agent [5]. Thus, identification of the underlying etiology in secondary AIHA is crucial, as it guides therapeutic selection, improves disease control, and reduces the risk of relapse. Patients with cold agglutinin activity should be advised to avoid cold environments, as cold exposure may precipitate hemolytic crises [15]. Chronic hemolysis is also associated with hypercoagulability, increasing the risk of venous thromboembolism (VTE); hence, VTE prophylaxis should be considered in hospitalized patients [13,16]. This case underscores the diagnostic complexity and therapeutic challenges of mixed-type AIHA and highlights the importance of individualized transfusion strategies, early immunosuppressive intervention, and vigilant long-term follow-up for underlying systemic disease.

## Conclusions

Mixed-type AIHA is a rare but clinically significant condition that requires a high index of suspicion due to its overlapping serologic features and potential for rapid, life-threatening anemia. Effective management necessitates a multidisciplinary approach, encompassing careful transfusion strategies with least-incompatible, antigen-matched red blood cells, prompt initiation of immunosuppressive therapy, and vigilant monitoring for hemolytic and thromboembolic complications. Recognition of an underlying systemic autoimmune disorder is crucial, as early diagnosis can guide long-term management and improve patient outcomes. This case underscores the importance of individualised therapy, awareness of dual-pathway hemolysis, and close follow-up to mitigate complications and optimise recovery.
